# Supplementary material for: Causal Relationship between Aging and Anorexia Nervosa: A White-Matter-Microstructure-Mediated Mendelian Randomization Analysis
Source: Biomedicines. 2024 Aug 16;12(8):1874. doi: 10.3390/biomedicines12081874 (PMC11351342; doi:10.3390/biomedicines12081874)
Supplement: Supplementary file 1 [file biomedicines-12-01874-s001.zip › Supplementary Figure/Figure S4/Singe SNP plot of MR analysis for TL to ubm-b-1641.pdf]

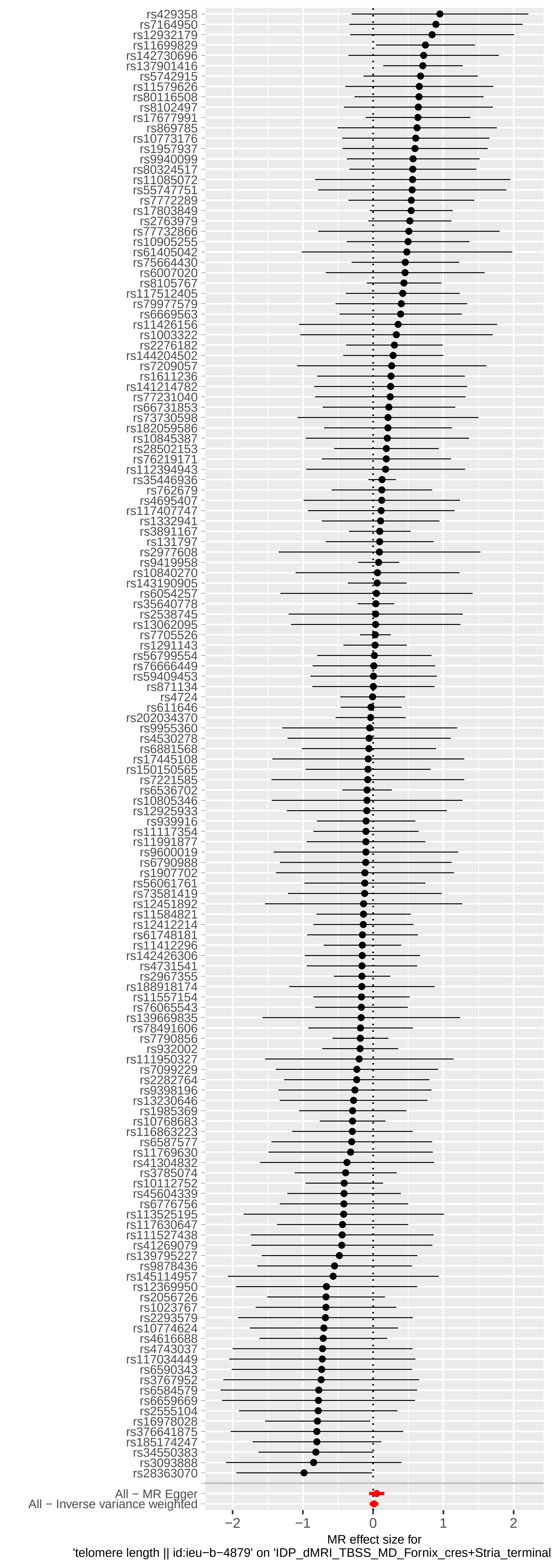
rs429358
rs7164950
rs12932179
rs11699829
rs142730696
rs137901416
rs5742915
rs11579626
rs80116508
rs8102497
rs17677991
rs869785
rs10773176
rs1957937
rs9940099
rs80324517
rs11085072
rs55747751
rs7772289
rs17803849
rs2763979
rs77732866
rs10905255
rs61405042
rs75664430
rs6007020
rs8105767
rs117512405
rs79977579
rs6669563
rs11426156
rs1003322
rs2276182
rs144204502
rs7209057
rs1611236
rs141214782
rs77231040
rs66731853
rs73730598
rs182059586
rs10845387
rs28502153
rs76219171
rs112394943
rs35446936
rs762679
rs4695407
rs117407747
rs1332941
rs3891167
rs131797
rs2977608
rs9419958
rs10840270
rs143190905
rs6054257
rs35640778
rs2538745
rs13062095
rs7705526
rs1291143
rs56799554
rs76666449
rs59409453
rs871134
rs4724
rs611646
rs202034370
rs9955360
rs4530278
rs6881568
rs17445108
rs150150565
rs7221585
rs6536702
rs10805346
rs12925933
rs939916
rs11117354
rs11991877
rs9600019
rs6790988
rs1907702
rs56061761
rs73581419
rs12451892
rs11584821
rs12412214
rs61748181
rs11412296
rs142426306
rs4731541
rs2967355
rs188918174
rs11557154
rs76065543
rs139669835
rs78491606
rs7790856
rs932002
rs111950327
rs7099229
rs2282764
rs9398196
rs13230646
rs1985369
rs10768683
rs116863223
rs6587577
rs11769630
rs41304832
rs3785074
rs10112752
rs45604339
rs6776756
rs113525195
rs117630647
rs111527438
rs41269079
rs139795227
rs9878436
rs145114957
rs12369950
rs2056726
rs1023767
rs2293579
rs10774624
rs4616688
rs4743037
rs117034449
rs6590343
rs3767952
rs6584579
rs6659669
rs2555104
rs16978028
rs376641875
rs185174247
rs34550383
rs3093888
rs28363070
All − MR Egger
All − Inverse variance weighted
−2
−1
0
1
2
MR effect size for
'telomere length || id:ieu−b−4879' on 'IDP_dMRI_TBSS_MD_Fornix_cres+Stria_terminal
